# Ambergris

**Published:** 1876-11

**Authors:** 


Ambergris.—Ambergris is a morbid secretion of the liver of the spermacetti whale. It is found floating on the sea, or deposited on the coast in warm climates inhabited by these whales. When of good quality it is of a bright gray color, streaked with black and yellow, and so soft that it may be flattened in the fingers. Its fracture presents a fine grain, and upon being cut its surface presents a waxy appearance. It exhales a fragrant odor when heated, a peculiarity which the counterfeit does not present.—*Ibid.*

